# Intestinal metastasis from choriocarcinoma: a case series and literature review

**DOI:** 10.1186/s12957-022-02623-0

**Published:** 2022-06-01

**Authors:** Yuting Wang, Zhe Wang, Xiaoxu Zhu, Qihong Wan, Peilin Han, Jun Ying, Jianhua Qian

**Affiliations:** Department of Gynaecology, The First Affiliated Hospital, Zhejiang University School of Medicine, Hangzhou, Zhejiang China

**Keywords:** Choriocarcinoma, Intestinal metastasis from choriocarcinoma, Case series, Chemotherapy, Diagnosis

## Abstract

**Background:**

Gestational choriocarcinoma is a rare trophoblastic tumor that spreads mainly to the lung, liver, and central nervous system. Fewer than 5% of patients present with metastasis to the gastrointestinal system and have a poor prognosis

**Case presentation:**

We describe four cases of patients with intestinal metastasis from choriocarcinoma who visited the First Affiliated Hospital of Zhejiang University School of Medicine and the First People’s Hospital of Hangzhou between April 2012 and October 2019. Four patients presented with gastrointestinal symptoms or developed gastrointestinal symptoms during treatment for choriocarcinoma. Three patients had these intestinal lesions surgically removed, and the postoperative pathology results suggested choriocarcinoma. All patients received multiple chemotherapy regimens during treatment for suboptimal human chorionic gonadotropin (hCG) levels; one patient died 22 months after a definitive diagnosis was made, and the other three patients are still undergoing regular follow-up.

**Conclusion:**

Given the low incidence of intestinal metastases from choriocarcinoma, the metastatic route of intestinal metastases from choriocarcinoma remains to be elucidated, and diagnosis mainly depends on pathology findings. An effective treatment has not been determined, and surgical excision with chemotherapy is generally accepted.

## Introduction

Choriocarcinoma is a malignant disease characterized by the proliferation and interstitial transformation of abnormal chorionic trophoblast cells, the absence of a chorionic structure, and hemorrhage and necrosis. It occurs in 1 in 40,000 pregnancies in North America and Europe, but in southeast Asia, the incidence can reach up to 9.2 [1]. Choriocarcinoma can be divided into two categories: gestational and non-gestational. When choriocarcinoma is present as a component of a germ cell tumor or is associated with somatic mutations in hypofractionated carcinomas, it is called non-gestational choriocarcinoma [2, 3]. Most choriocarcinomas originate during pregnancy, which is called gestational choriocarcinoma, with more than 50% arising from a hydatidiform mole pregnancy, 25% associated with a full-term pregnancy or preterm delivery, and the remainder associated with miscarriage or tubal pregnancy. And the tubal pregnancy may lead to gestational tubal choriocarcinomas with an incidence of about 1.5/100,0000 [4].

Choriocarcinoma is extremely malignant, with early and extensive metastasis. It can spread distally to the lung, liver, and central nervous system, but fewer than 5% of patients show metastasis to the gastrointestinal system (with metastasis to the intestine being even rarer) and have a poor prognosis [5, 6]. Patients who have developed metastasis often present with symptoms of metastases. Considering that gestational choriocarcinoma and non-gestational choriocarcinoma have different genetic origins and levels of immunogenicity [7], their sensitivity to chemotherapy and treatment protocols also differ. Therefore, it is necessary to consider whether intestinal metastasis from gestational choriocarcinoma occurs when these patients present with gastrointestinal symptoms combined with hCG elevation.

Knowledge of the symptoms, metastatic pathways, and differential diagnosis of intestinal metastasis from choriocarcinoma can help clinicians identify these patients at an early stage. In this paper, we describe four cases of patients with intestinal metastasis from choriocarcinoma who visited the First Affiliated Hospital, Zhejiang University, and the First People’s Hospital of Hangzhou between April 2012 and October 2019 and then summarize the characteristic symptoms of the disease through a review of the literature to provide insights into the early diagnosis and differential diagnosis of this disease.

## Case description

### Patient A

The patient was a 29-year-old female, G3P2, whose last pregnancy resulted in a full-term baby delivered by cesarean section in 2014 due to breech position and 1 week of umbilical cord entanglement. In 2013, she underwent curettage because of a hydatidiform mole, and her hCG level was in the normal range during regular follow-up. In March 2019, the patient went to Yiwu Central Hospital for follow-up, and her hCG level was 17,660 IU/mL. After monitoring her hCG level for 2 weeks, it remained elevated, at 23,315 IU/mL. Transvaginal color Doppler ultrasound (TVS) showed a heterogeneous hypoechoic mass in the left ovary, indicative of ectopic pregnancy. Laparoscopic exploration and hysteroscopy were performed at the local hospital, but no obvious lesion was found, so she was referred to our hospital.

**Fig. 1:**
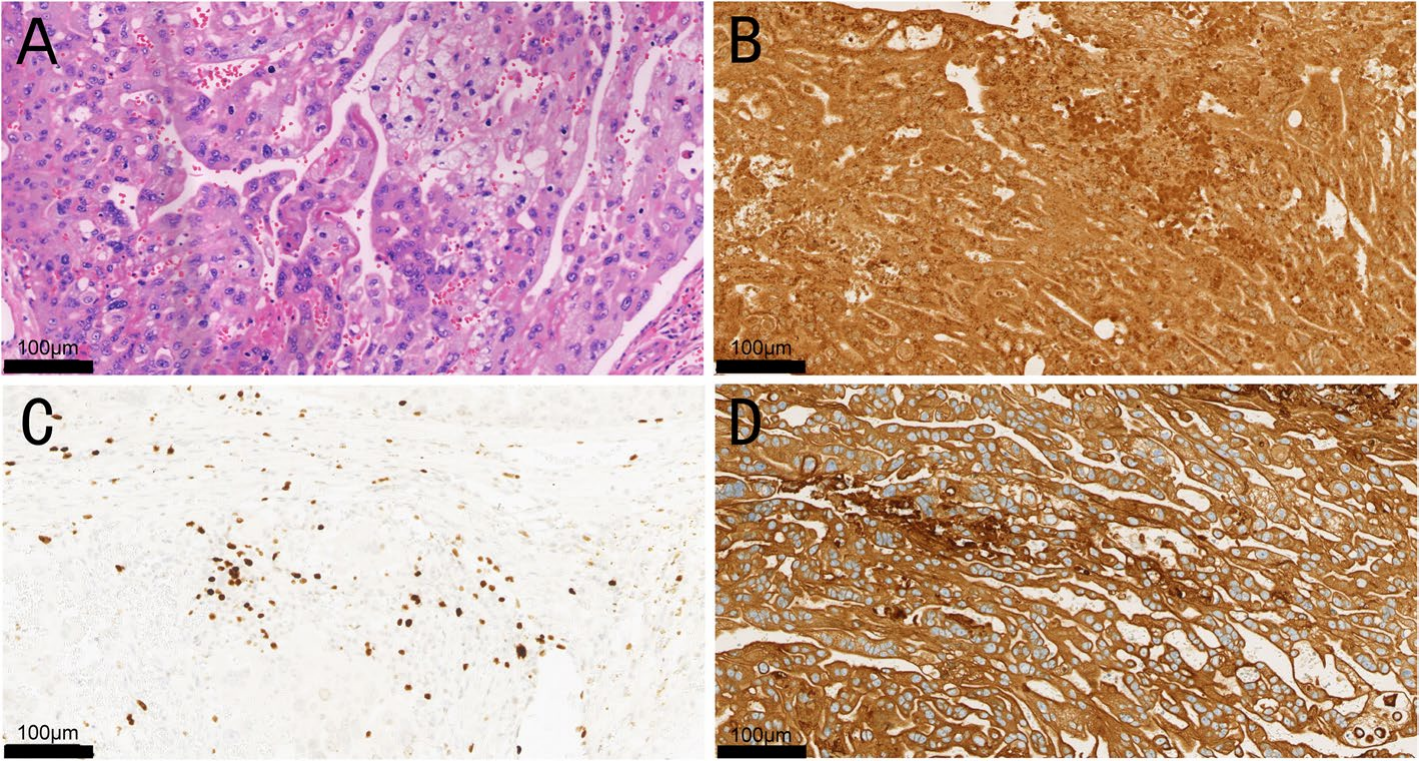
The pathology and immunohistochemistry of patient A. Hematoxylin and eosin (HE) staining shows the infiltrative growth of tumor cells in the form of thin beams and nested clusters involving the mucosa and submucosa of the small intestine, with obvious tumor cell heterogeneity, pleomorphism, vacuolation of some cells, clearly visible nucleoli, and regional lamellar necrotic changes. Immunohistochemistry indicating that the tumor is positive for CK (pan), Ki-67, and β-hCG. The Ki-67 index is about 25% ± 5%. **A** HE staining, 20× magnification. **B** Immunostaining for CK (pan), 20× magnification. **C** Immunostaining for Ki-67, 20× magnification. **D** Immunostaining for β-hCG, 20× magnification

**Fig. 2:**
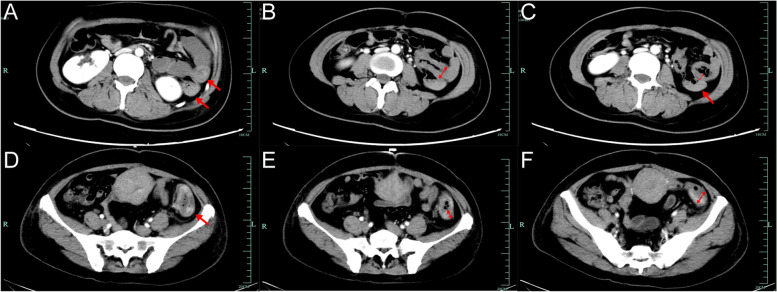
The follow-up CT scan of patient A. **A** Small bowel junction in plain CT scan (red arrow). **B** Small bowel junction in enhanced CT scan (red arrow). **C** Several calcifications within the liver and no obvious metastases in enhanced CT scan. **D** No obvious lesions within uterus in enhanced CT scan

On admission, the patient reported feeling dizzy and weak with nausea and vomiting and had reddish-brown stools but no significant vaginal bleeding. Pelvic examination was unremarkable. Laboratory tests revealed the following: hCG 33,718 mIU/mL, Hb 107 g/L, and CA125 31.9 U/mL and a weakly positive fecal occult blood test. TVS showed echogenic changes in the muscle layer of the left anterior wall. Whole-abdomen CT showed a patchy hypodense shadow in the uterus, a slightly thickened small bowel wall in part of the left mid abdomen, a slightly dense shadow in part of the small bowel and colorectal lumen (Fig. 4), and hypodense foci in the S4 segment of the liver. CT of the lung showed a nodule in the right middle lobe of the lung at the cardio-diaphragmatic angle with free gas under the diaphragm. The patient was diagnosed with choriocarcinoma (IV:14). The patient’s Hb level was 107 g/L at admission, and she was administered the EMA chemotherapy regimen on the same day. Two days later, the patient developed abdominal distention with nausea and vomiting, and her blood pressure was approximately 80/50 mmHg. Her Hb level was 47 g/L when checked and continued to fall to 40 g/L even after a 1.5-unit infusion of suspended red blood cells, and she had a strongly positive fecal occult blood test. Considering that the intestinal lesion continued to bleed, an emergency operation was performed, and part of her small intestine was resected. Intraoperatively, a mass approximately 3 cm in diameter was palpated in the intestine 45 cm from the flexural ligament. The mass was microscopically observed to show the infiltrative growth of tumor cells in the form of thin beams and nested clusters involving the mucosa and submucosa of the small intestine, with obvious tumor cell heterogeneity, pleomorphism, vacuolation of some cells, clearly visible nucleoli, and regional lamellar necrotic changes. Immunohistochemistry indicated that the tumor was positive for CK (pan), β-hCG, HPL, Ki-67, CD2, CD3, CD5, CD7, CD8, desmin, and SMA and partially positive for CD4 and CD20 (Fig. 1). Postoperatively, the patient underwent 1 high-dose cycle of EMA-CO, which decreased her hCG level to 15.7 mIU/mL, followed by 7 conventional cycles of EMA-CO. No significant new lesions were detected on CT of the lung or whole-abdomen CT at regular follow-ups (Fig. 2). The patient is still being monitored regularly.

### Patient B

**Fig. 3:**
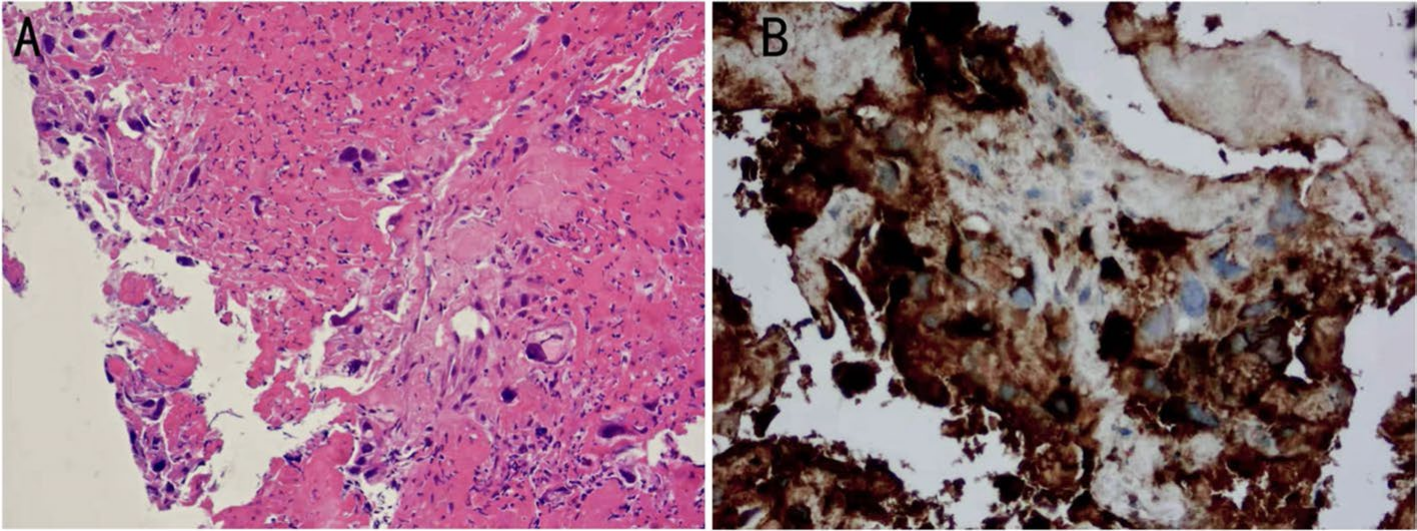
The biopsy pathology and immunohistochemistry of Patient B. HE staining shows some heterotypic cells with hyperchromatic nuclei in denatured necrotic structures. The β-hCG is positive for the heterotypic cells, which are considered as syncytiotrophoblast cells. **A** HE staining, 10× magnification. **B** Immunostaining for β-hCG, 40× magnification

The patient was a 46-year-old female, G3P2, whose last pregnancy resulted in a full-term baby delivered by cesarean section in 2003 due to uterine scarring; she denied a history of molar pregnancy. The patient presented to the Women Hospital School of Medicine Zhejiang University in January 2018 with vaginal bleeding for more than 40 days and right lower abdominal pain with nausea and vomiting for more than 30 days. Laboratory tests suggested an hCG level greater than 90,000 U/L. TVS showed an enlarged uterus at 40 days of gestation and a 4.4 × 3.2 × 2.5 cm inhomogeneous echogenicity in the uterine cavity with blood flow inside, poor demarcation from the anterior muscular wall, and cystic masses in both adnexal areas. Computed tomography (CT) of the whole abdomen indicated multiple intrahepatic occupancies, and the intestinal wall was thickened at the junction of the sigmoid colon and descending colon. Colonoscopy was performed at the Affiliated Hospital of Hangzhou Normal University and biopsy pathology combined with history implied intestinal metastasis from choriocarcinoma (Fig. 3).

**Fig. 4:**
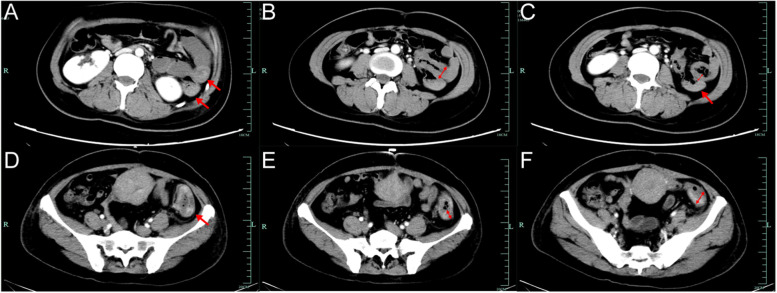
The whole-abdomen CT images: lesions in the intestine presented as thickening of the bowel wall and blood in the intestine presented as slightly dense shadows in the intestine. Patient A: **A** a slightly dense shadow in part of the small bowel and colorectal lumen (red arrow), **B** a slightly thickened small bowel wall in part of the left mid abdomen in the coronal section (red double arrow), and **C** a slightly thickened small bowel wall in the transverse section (red double arrow) and a slightly dense shadow in part of the small bowel (red arrow). Patient B: **D** thickening of the bowel wall in the lower part of the descending colon (red arrow), **E** long diameter of the thickened portion of the bowel (red double arrow), and **F** transverse diameter of the thickened portion of the bowel (red double arrow)

At the time of presentation at our hospital, the patient had a small amount of vaginal bleeding and reported nausea and vomiting but no significant abdominal pain. Physical examination showed tachycardia and signs of anemia, such as pallor of the face and lips. Pelvic examination showed a small amount of bloody vaginal discharge and no other abnormalities. Laboratory tests showed the following: hCG 70,626 mIU/mL, hemoglobin (Hb) 58 g/L, carbohydrate antigen 125 (CA125) 46.6 U/ml, carcinoembryonic antigen (CEA) 5.7 ng/mL, and a weakly positive fecal occult blood test. Whole-abdomen CT showed thickening of the bowel wall in the lower part of the descending colon (Fig. 4), along with peri-intestinal and retroperitoneal lymph node metastases. CT of the lung showed multiple nodules in the right lung and an enlarged mediastinal multiple lymph node shadow. In conjunction with the patient’s history, she was diagnosed with choriocarcinoma (IV:19). After admission, she was administered 5 cycles of EMA/CO (etoposide, methotrexate, actinomycin D, cyclophosphamide, vincristine), 1 cycle of FAEV (floxuridine, dactinomycin, etoposide, vincristine), 1 cycle of paclitaxel + oxaliplatin + gemcitabine, and 7 weekly cycles of TEP (paclitaxel, etoposide, cisplatin) chemotherapy. Due to the repeat elevation of hCG levels, laparotomy was performed, and the uterus, fallopian tubes and ovaries, and partial colon were resected successfully. Intraoperative dissection of the intestine showed local ulcer-like changes in the mucosa. The resected specimen showed a microscopically heterogeneous, glandular, nest-like arrangement of tumor cells with an infiltrative growth pattern and invasion of the muscular layer. Immunohistochemistry indicated that the tumor was strongly positive for P53; positive for CK, Ki-67, and EMA; partially positive for CK7, CK20, and AFP; and partially weakly positive for CDX2. Postoperatively, 5 cycles of BEP (bleomycin, etoposide, cisplatin) (4 incomplete), 2 cycles of MBE (methotrexate, bleomycin, etoposide), 2 cycles of EP/EMA (etoposide, cisplatin, methotrexate, actinomycin D), and 8 cycles of pembrolizumab were administered. The patient eventually died of respiratory failure due to multiple metastases in the lungs 22 months after diagnosis.

### Patient C

The patient was a 36-year-old female, G2P2, whose last pregnancy resulted in a full-term baby delivered by cesarean section in 2018 due to uterine scarring. She presented to Ningbo Hospital of Zhejiang University in September 2019 because of abnormal intermenstrual vaginal bleeding for 3 months and sudden dizziness and headache for 12 h. Laboratory tests showed her hCG level was 29,387 mIU/mL, and TVS showed no obvious pregnancy tissue in the uterus. Cranial CT showed cerebral hemorrhage in the left occipitoparietal lobe and a small amount of subarachnoid hemorrhage. The left parietooccipital lobe tumor was resected, and the postoperative pathology suggested brain metastasis or invasive malignancy; the lesion characteristics were consistent with metastatic choriocarcinoma. Postoperatively, the patient reported blood in her stool several times, so her hCG was retested and found to be 112,477 mIU/mL. Whole-abdomen CT showed a slightly dilated colon with effusion, an occupancy in the right para-pelvis, a lack of blood supply foci in the spleen, and possible infarction. Mesenteric computed tomographic angiography (CTA) suggested bleeding in the small intestine, and emergency dissection was performed. Microscopically, the tumor cells showed infiltration into the entire intestinal wall, with pulsatile carcinoma thrombus positivity and no obvious nerve invasion.

The patient’s vital signs were unstable at the time of presentation at our hospital. Physical examination shows the pallor of the face. Laboratory tests revealed the following: hCG 71,990 mIU/mL, Hb 68 g/L, and CA125 79.9 U/mL. The lung CT revealed a right pulmonary nodule, and metastases were considered in the context of the patient’s medical history. Whole-abdomen CT showed a dilated uterine cavity with an enhancing nodule in the lower uterine cavity, and CT revealed the presence of a right suprarenal pole occupancy, anterior median abdominal wall nodules, and pelvic floor nodules, all of which were considered metastases. Pathology consultation was performed in our hospital, and immunohistochemistry of the abovementioned intestinal mass showed positivity for CK and β-hCG and scattered positivity for HPL, P40, and P63. Combined with the external medical history, the patient was diagnosed with choriocarcinoma (IV:16). Upon admission, she was treated with anti-infectives, gastric secretion inhibitors, gastric protectants, platelets, erythropoiesis support, and parenteral nutrition. After her vital signs had stabilized, the patient received 6 cycles of high-dose EMA-CO, 2 cycles of FAEV, and 12 weekly cycles of TEP at our hospital. Late monitoring of hCG showed that her levels were within the normal range. The patient is still being monitored regularly.

### Patient D

The patient was a 26-year-old female, G1P1, whose last pregnancy resulted in a full-term baby delivered by cesarean section in 2012 due to hypertension. In April 2012, the patient visited the First People’s Hospital in Hangzhou for vaginal bleeding 51 days after delivery and persistent high temperature. Pelvic examination suggested thickening of the bilateral adnexal area without obvious pressure pain. Laboratory tests showed the following: hCG 357.2 mIU/mL, C-reactive protein (CRP) 80 mg/L, Hb 79 g/L, and a strongly positive fecal occult blood test. The patient was administered anti-infective treatment, but it was not effective. Seven days later, her hCG level was > 1,000,000 mIU/mL, estradiol level was > 3670 pmol/L, and CEA level was 11.96 μg/L. Various imaging studies were performed, and upper abdominal ultrasound revealed multiple nodules in the liver. TVS showed a postpartum uterus, gross endometrium, and cystic masses in both ovaries. PET-CT revealed a nodule of approximately 10 mm in diameter in the posterior basal segment of the lower lobe of the right lung. In addition, a nodule-like concentration was observed in the left epigastric small intestine. In combination with the medical history, choriocarcinoma with extensive metastases to the liver, lung, and small intestine was considered. The patient was subsequently diagnosed with choriocarcinoma (IV:16). After a definite diagnosis, the patient did not undergo surgery for small bowel tumors but was instead administered 5 cycles of EMA-CO and 3 cycles of EP-EMA. However, due to the slow decline in her hCG level, the chemotherapy regimen was changed to 2 cycles of PEB and 1 cycle of FAEV. The liver lesions gradually shrank during follow-up, and there were no obvious symptoms of gastrointestinal bleeding. The patient is now in stable condition and is being monitored regularly.

**Fig. 5:**
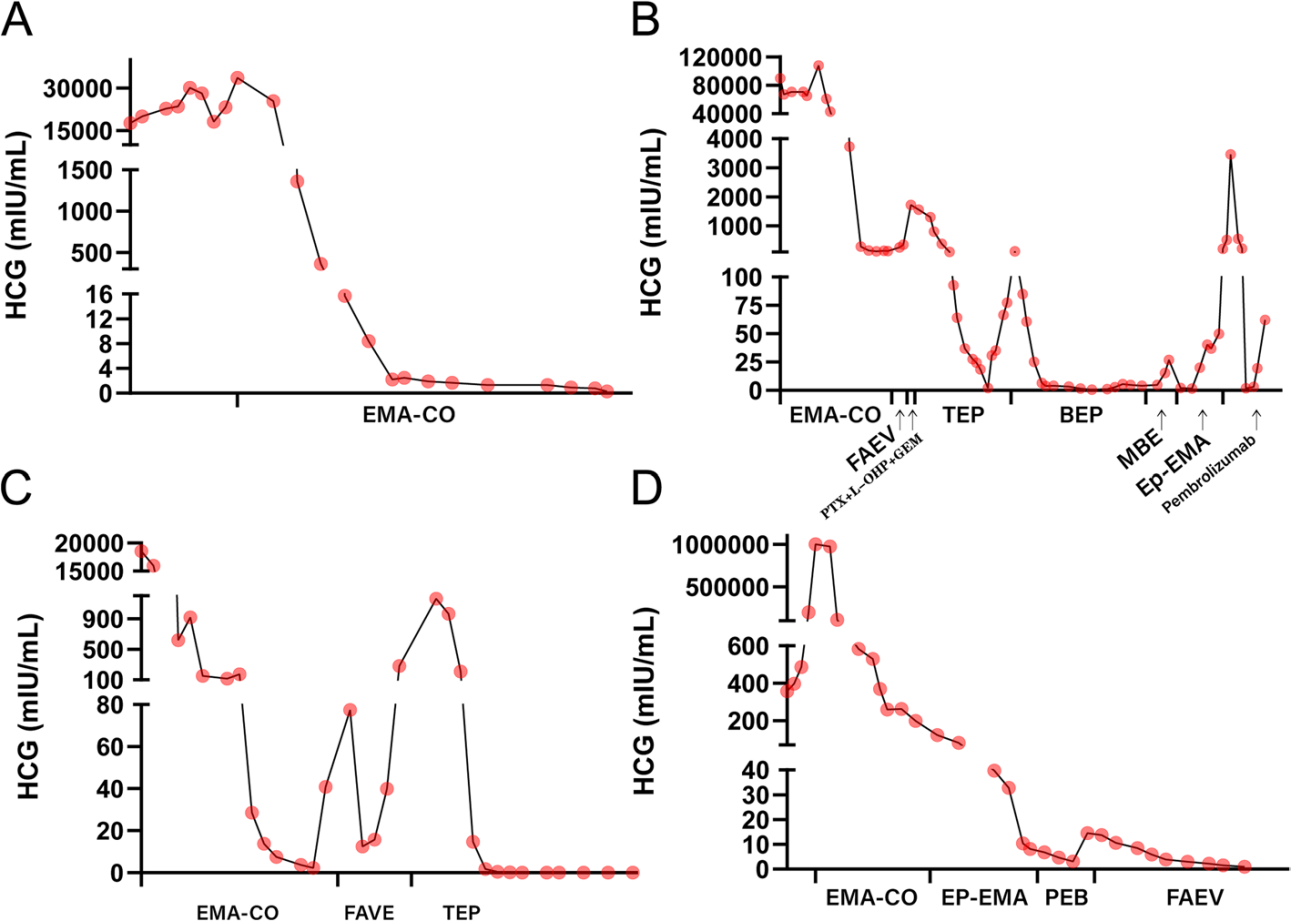
Trends in hCG changes and corresponding treatment regimens in four patients. **A** Patient A (IV: 14) was treated with the EMA-CO regimen, and hCG levels decreased to normal and remained stable. **B** Patient B (IV: 19) changed chemotherapy regimens successively to FAVE, paclitaxel + oxaliplatin + gemcitabine, TEP, BEP, MBE, EP-EMA, and pembrolizumab due to unsatisfactory hCG levels decreases. **C** Patient C changed chemotherapy regimens successively to FAVE and TEP due to recurrent elevated hCG levels. **D** Patient D changed chemotherapy regimens successively to EP-EMA, PEB, and FAEV due to unsatisfactory hCG level decreases

The trends in hCG changes, and corresponding treatment regimens administered to these four patients are presented in Fig. 5. A summary of the characteristics of the four patients is listed in Table 1.

**Table 1:** Summary of the characteristics of the four cases

	Age	Symptom	Time until last pregnancy	hCG level before treatment (IU/mL)	Hb level before treatment (g/L)	Tumor markers before treatment (ug/L)	Primary uterine lesion	Metastatic lesion	Treatment	Outcome
Patient A	29 y	melanorrhagia	5 years	17,660	107	CEA: 1.3 CA125: 31.9	Yes	Small intestine, lung, liver	Small bowel lesion resection and anastomosis, chemotherapy	Alive
Patient B	46 y	Vaginal bleeding, right lower abdominal pain, nausea and vomiting	15 years	> 90,000	63	CA125: 46.6	Yes	Colon, lung, liver	Colon lesion resection and anastomosis, hysteroscopy, chemotherapy	Died 22 months after diagnosis
Patient C	36 y	melanorrhagia	11 months	112,477	79	CEA: 1.4 CA125: 79.9	Yes	Small intestine, lung, liver, kidney, brain	Small bowel lesion resection and anastomosis, chemotherapy	Alive
Patient D	26 y	melanorrhagia	51 days	> 1,000,000	79	CEA: 11.96	No	Small intestine, lung, liver	Chemotherapy	Alive

## Discussion and conclusions

Intestinal metastases from choriocarcinoma are rare, occurring in only approximately 5% of patients with choriocarcinoma; lower gastrointestinal metastases are even rarer, but intestinal metastases often predict a poor prognosis [8, 9]. This disease was first described by Sears JB et al. in 1933, and only 27 cases have been reported to date in the English literature (partially shown in Table 2 [5, 10–19]). In the early reported cases, limitations in the understanding of gestational trophoblastic neoplasms and ancillary examination techniques precluded a definitive diagnosis in most cases. The diagnostic difficulty is further increased when the patient has no clear gynecological symptoms, such as vaginal bleeding, or when the symptoms of tumors at the primary site are masked by those at metastatic sites. In this paper, we discuss the features of choriocarcinoma such as the clinical features, treatment, and differential diagnosis.

**Table 2:** A compilation of reported cases of intestinal metastases from choriocarcinoma published in English from 1933 to the present

	Age	Symptoms	Time until last pregnancy	hCG level before treatment (IU/mL)	Metastatic lesions	Primary uterine lesion	Treatment	Outcome
Bin Chet Toh (2020) [10] Fatemi SR (2017) [11]	38 y 33 y	melanorrhagia Abdominalgia, melanorrhagia	Not available 5 years	6,764 57,000	Jejunum, lung Jejunum	No Yes	Selective coil vascular embolization, chemotherapy Jejunum lesion resection and anastomosis, chemotherapy	Not available Alive
En Bee Cho (2016) [5]	40 y	Abdominalgia, vaginal bleeding	2 years	8,837	Jejunum, lung, liver	No	Jejunum lesion resection and anastomosis, diagnostic curettage, EMA-CO	Alive
Anisha Ramessur (2015) [12]	32 y	melanorrhagia, anemia	5 years	77,000	Jejunum, lung, liver	YES	EP chemotherapy	Alive
Anil Arora (2013) [13]	31 y	Lower GI bleeding	5 months	25,000	Proximal ileum, cecum, ascending colon, sigmoid, rectum	Yes	Ileal lesion resection and anastomosis	Not available
Bokhari (2009) [14]	35 y	Lower GI bleeding	1 month	Not available	Terminal ileum, lung, spleen, kidney, posterior mediastinum	No	Ileal lesion resection and anastomosis	Not available
M Chaturvedi (2005) [15]	30y	Weakness, fatiguability, giddiness, breathlessness, and palpitations	6 months	Not available	Ileum, descending colon, lung, liver, pancreas, right kidney	No	Not available	Died 5 days after admitted to hospital
PG Balagopal (2003) [16]	32 y	Abdominalgia, vomiting	Not available	Not available	Jejunum, lung, liver, brain	No	Small bowel resection and anastomosis, EMA-CO/BEP	Not available
Bina Ravi (1997) [17]	22 y	Fever, vomiting, abdominal distention	Not available	Not available	Small bowel, liver, spleen	No	Small bowel resection and anastomosis	Died 8 days after surgery
I.T. Magrath (1971) [18]	23y	melanorrhagia	1 month	> 1,000,000 IU/day	Proximal ileum, ampulla of Vater, lung, liver	No	Small bowel resection and anastomosis	Died 7 months after treatment
Sear JB (1933) [19]	32y	melanorrhagia, weakness	3 years	Not available	Jejunum, omentum, liver	No	Small bowel resection and anastomosis, X-ray treatment	Died 1 month after surgery

### Clinical features, pathological examination, and immunohistochemistry

Intestinal metastases from choriocarcinoma are most often found in fertile women with a history of pregnancy, and they typically visit the hospital for 4 reasons: (1) upper gastrointestinal bleeding, i.e., vomiting blood; (2) lower gastrointestinal bleeding, i.e., blood in stool, black fecal syndrome, or routine stool examination suggesting positive occult blood; (3) intractable anemia, i.e., malaise and an anemic appearance, with intestinal masses found during ancillary examinations; and (4) acute abdomen, with common causes including abdominal internal bleeding and intestinal obstruction and less common causes including intussusception, intestinal perforation, and intractable anemia [20–22]. Patients with intestinal metastases from choriocarcinoma often have obvious signs of anemia at the time of presentation, such as pallor of the face, lips, lid conjunctiva, and nail bed, and complete blood count results suggest a significant decrease in hemoglobin. Thus, the hCG level in combination with imaging assessments can play an important role in the diagnosis of this disease. Whole-abdomen CT is most commonly used to assess the presence of intestinal lesions, which may present as thickening of the intestinal wall or a soft tissue shadow in the intestine, but previous case reports did not describe the presence of lymph node enlargement. The lesion appears as a markedly enhanced nodular shadow on mesenteric vascular CTA, and a large amount of contrast leakage suggests the presence of active bleeding [10].

Pathology is the gold standard for diagnosis, and it can be clearly diagnosed by pathological examination of specimens excised by gastroscopy, colonoscopy, or surgery in combination with hCG levels. Gastroscopy reveals polypoid lesions or multiple ulcerated lesions of variable size in the intestine with a white fragile base that can bleed easily when touched. They are most commonly found in the jejunum, followed by the duodenum and ileum. Microscopically, patches of heterogeneous proliferating trophoblast cells are observed infiltrating the surrounding tissues and blood vessels in a glandular, nest-like arrangement, and most of the tumor cells show biphasic differentiation, with a close mixture of cytotrophoblast and syncytial trophoblast cells [1]. The depth of the intestinal lesions can reach the mucosa, submucosa, and some muscle layers [13].

Immunohistochemistry occupies an important position in the differential diagnosis of relevant diseases. Ki-67, as an oncogene, is expressed in cells at different proliferative stages. Therefore, Ki-67 is mostly used to reflect the degree of cell proliferation in clinical diagnosis. Previous studies have reported that the average Ki-67 marker index for gestational choriocarcinoma is 69 ± 20%, that for intermediate trophoblast it is about 18 ± 5%, and that for placental site trophoblastic tumors is 14 ± 6.9 %[23]. SALL4 and PLAP are commonly used for the primary screening of germ cell tumors, among which SALL4 is expressed in both germ cell and gestational choriocarcinoma [24, 25]. The most commonly used protein marker for trophoblastic tumor confirmation is β-hCG, followed by PD-L1, inhibin, and GATA-3 [26]; however, none of these markers can distinguish the histological origin of the tumor [27, 28]. Choriocarcinoma often presents broad-spectrum keratin positivity, with 100% of choriocarcinomas presenting CK7 positivity, 87% presenting inhibin positivity, and 85% presenting p63 positivity [29]. Because choriocarcinomas are prone to bleeding, biopsy at the lesion site carries a high risk of life-threatening and persistent bleeding; therefore, the European Society for Medical Oncology (ESMO) guidelines emphasize that biopsy is not necessary before starting chemotherapy [30], which places limitations on the ability to obtain a definitive diagnosis.

### Treatment

Given the low incidence of intestinal metastases from choriocarcinoma, there is no consensus on treatment options at this time. Common treatment options include surgery and chemotherapy, with chemotherapy being the cornerstone of treatment. Surgery can be performed to relieve symptoms, improve anemia, and stabilize vital signs. In a review of previous cases, the main surgical approach was partial bowel resection and end-to-end anastomosis at the site of the lesion. However, for patients with severe anemia and extremely unstable vital signs, the surgical extent can be greatly limited, and recovery from surgery may delay the administration of chemotherapy and lead to progression of the remaining metastatic lesions. Toh BC et al. performed selective coil vascular embolization of the mid jejunal branches of the superior mesenteric artery to achieve hemostasis in a patient with an actively bleeding jejunal lesion, and a late review did not reveal rebleeding of the intestinal lesion or intestinal ischemia [10]. Given the high sensitivity of choriocarcinoma to chemotherapy, Anisha Ramessur et al. administered a low-dose EP chemotherapy regimen (etoposide 100 mg/m^2^ and cisplatin 20 mg/m^2^) to reduce bleeding and side effects due to chemotherapeutic drug metabolism in patients with intestinal metastases from choriocarcinoma, causing intussusception and bleeding from intestinal lesions. Repeat CT 6 days after the initiation of chemotherapy suggested the complete resolution of intussusception [12]. Patient D in this paper also achieved complete remission of the intestinal lesions by chemotherapy with EMA-CO, EP-EMA, PEB, and FAEV regimens. As the medical field continues to advance, new treatment modalities continue to be explored and applied, but due to the lack of statistical data, a traditional surgical approach is still an important part of treatment for intestinal metastatic lesions from choriocarcinoma.

### Metastasis

Choriocarcinoma is characterized by early metastasis. Existing studies suggest that the mode of metastasis from choriocarcinoma is mainly hematogenous, and the liver is the most common site of abdominal metastasis [31]. To date, the route of intestinal metastasis from choriocarcinoma has not been clearly described. Bina Ravi et al. found that when intestinal metastasis from choriocarcinoma occurred, patients inevitably had a combination of pulmonary and hepatic metastases [17], with no evidence of lymph node metastases [16]. A similar phenomenon can be observed in Tables 1 and 2; in this series, the metastatic lesions mostly invaded the muscular layer of the intestine. Thus, in terms of the metastatic pathway, we hypothesize the following: (1) the primary uterine lesion metastasized to the liver hematologically and then metastasized to the intestine hematologically. (2) The primary uterine lesion first metastasized to the liver hematologically, then metastasized to the greater omentum via implantation, and finally metastasized to the intestine hematologically via the greater omentum. In a review of previous case reports, the greater omentum was often found to be involved at the time of dissection, but the metastatic sequence between the greater omentum and the intestine was unclear. (3) The primary uterine lesion metastasized to the greater omentum via implantation and then to the liver and intestine hematologically. Further study of the metastatic pathway can provide guidance for the adjuvant workup of patients who have already been diagnosed or have yet to be definitively diagnosed with choriocarcinoma. Although most patients at high risk of metastasis first experience symptoms or undergo imaging that reveals pulmonary metastases, a comprehensive evaluation including whole-abdomen CT is recommended for all patients, not only those with pulmonary metastases [31].

### Differential diagnosis

Intestinal metastases from gestational choriocarcinoma need to be differentiated from non-gestational choriocarcinoma, which mainly includes primary intestinal choriocarcinoma and intestinal metastases from hCG-secreting ovarian germ cell tumors.

The prognosis of primary intestinal choriocarcinoma is poor, with only 31 cases reported to date. Patients with intestinal metastases from gestational choriocarcinoma and primary intestinal choriocarcinoma differ in terms of the genetic origin of the tumor, immunogenicity, sensitivity to chemotherapy, and prognosis. Intestinal metastases from gestational choriocarcinoma are usually regarded as allogeneic grafts with strong immunogenicity, a better response to cytotoxic drugs, sensitivity to chemotherapy, and good prognosis. In primary intestinal choriocarcinoma, the tumor tissue originates entirely from the patient, has no allogeneic components, is weakly immunogenic, and is easily resistant to drugs, requiring the selection of surgical procedures and the combination of multidrug chemotherapy according to the stage of the disease [32]. Both can present with elevated hCG levels, intestinal lesions on imaging and pathology indicative of trophoblastic infiltration, and hemorrhagic necrosis, suggesting a lack of specific biomarkers, making clinical differential diagnosis difficult. Primary intestinal choriocarcinoma should be highly suspected in patients who are sexually immature, unable to conceive, or have never had sex and who also have elevated serum hCG levels and intestinal lesion pathology suggestive of choriocarcinoma. Some researchers believe that an interval of 15 years or more between the last pregnancy and the detection of choriocarcinoma should be considered non-gestational choriocarcinoma [33, 34]. This remains controversial, as the interval between the last pregnancy and the diagnosis of choriocarcinoma was as short as 4 weeks and as long as 25 years in the cases collected by Mangla [20].

The following three methods can be used to differentially diagnose patients with a history of pregnancy.

#### The site of metastasis

Bina et al. found that patients with intestinal metastases from gestational choriocarcinoma inevitably have a combination of pulmonary and hepatic metastases [17]. However, a review of 31 reported cases of primary intestinal choriocarcinoma revealed that 14 cases had pulmonary metastases, and 23 cases had liver metastases, of which 8 cases had liver metastases only [35].

#### Pathological examination

In addition to choriocarcinoma components, primary choriocarcinoma usually contains other recognizable tissue components such as adenocarcinoma, clear cell carcinoma, squamous cell carcinoma, giant cell carcinoma, or urothelial carcinoma [36]. Several hypotheses have been proposed regarding the origin of primary gastric choriocarcinoma: (1) Davidsohn suggested that primary choriocarcinoma originates in the scattered gonadal region of the abdomen; (2) Koritschoner believed that it could be derived from long-term metastasis of choriocarcinoma in utero (up to 22 years); (3) Hartz et al. suggested that it originates from gastric teratomas; (4) Voss speculated that it originates from a potential protogonadal cell in gastric polyps; and (5) Pick indicated that the dedifferentiation of cancer cells to the level of embryonic ectoderm has the ability to form trophoblast cells, and choriocarcinoma is then generated through excessive growth and elimination of the original adenocarcinoma [37, 38]. The widely accepted theory of dedifferentiation proposed by Pick in 1926 is also applicable to explain the origin of primary intestinal choriocarcinoma. Among the 18 cases of primary intestinal choriocarcinoma reported in English, 14 cases were found to have the components of choriocarcinoma and adenocarcinoma under a microscope. There is usually a transition zone between the two tissue types, in which the adenocarcinoma component is composed of tall columnar cells. As the tumor spreads and metastases, the adenocarcinoma features gradually decrease, and the choriocarcinoma features gradually increase [1, 35, 39]. As such, liver metastasis from primary intestinal choriocarcinoma often only have choriocarcinoma components under a microscope. Histologically, the coexistence of choriocarcinoma with another malignant tumor (mixed germ cell tumor, adenocarcinoma, urothelial carcinoma, etc.) essentially denies the possibility of being relevant to pregnancy [40]. However, in simple choriocarcinoma or in cases where choriocarcinoma components dominate the tumor, it is not easy to determine the origin.

#### Genetic testing

A recognized feature for gestational choriocarcinoma is the presence of paternal genes, whereas non-gestational choriocarcinoma is of patient origin and does not have paternal genes. Thus, genetic testing of parent genes that are not in normal tissues can be clearly distinguished from intestinal metastasis from gestational choriocarcinoma and primary intestinal choriocarcinoma. Whaley RD et al. used fluorescence in situ hybridization to detect X and Y chromosome centromeres to determine whether they were from gestational choriocarcinoma [40]. In gestational choriocarcinoma, however, Y chromosome absence is possible regardless of paternal or parental origin. Fisher et al. applied DNA polymorphism analysis in the diagnosis of choriocarcinoma in 1992 in order to solve the problem [41]. Detecting a parent HLA antigen or conducting short array repeating sequence (STRS), gene analysis allowed for a clear diagnosis [24, 42, 43]. STR genotyping can be performed on formalin-fixed tissues and can provide interpretable results even in samples that have been fixed for a long time or are damaged. Therefore, STR genotyping shows promise in clinical applications and research. As detection sites increase, the accuracy of STR genotyping also increases.

The distinction between intestinal metastases from gestational choriocarcinoma and intestinal metastases from ovarian germ cell tumors is less challenging. Patients with germ cell tumors such as non-gestational choriocarcinoma of the ovary, malignant mixed germ cell tumors, asexual cell tumors, and yolk cystic tumors can have elevated β-hCG levels and are much less likely to exhibit intestinal metastases than those with ovarian epithelial tumors. Imaging examinations such as TVS and pelvic MRI are useful in detecting ovarian lesions. Malignant mixed germ cell tumors, asexual cell tumors, and yolk cysts can be clearly diagnosed using specimens excised by biopsy or surgery for pathological examination. There are no significant differences in histopathology and immunohistochemistry between non-gestational choriocarcinoma of the ovary and gestational choriocarcinoma [44], and a clear diagnosis can be obtained by testing for the presence of the paternal allele.

### Prognostic analysis

Gestational choriocarcinoma has a cure rate of 98% with chemotherapy, but after intestinal metastases occur, the prognosis is poor. A review of the literature and the cases presented herein indicates that early diagnosis and aggressive chemotherapy can significantly improve prognosis. The cooperation of a multidisciplinary team is important for the diagnosis of intestinal metastases from choriocarcinoma, but reliance on a department other than gynecology often leads to delayed diagnosis and bias in the choice of treatment. Another major reason for delayed diagnosis is that patients do not report their pregnancy history at the time of consultation or the time between the onset of clinical symptoms, and the last pregnancy is too long for the patient to recall the previous pregnancy; in the cases reviewed by Mangla M et al., the time between the last pregnancy and the diagnosis of choriocarcinoma was as short as 4 weeks and as long as 25 years [20]. In patients who have been diagnosed with choriocarcinoma, a thorough evaluation is including the intestine particularly important for the early detection of metastatic lesions.

Given the low incidence of intestinal metastases from choriocarcinoma, the characteristics of this disease remain to be explored. The above discussion will aid in the early diagnosis of intestinal metastases from choriocarcinoma. A thorough evaluation of patients with choriocarcinoma is essential. The metastatic route of intestinal metastases from choriocarcinoma remains to be elucidated. Currently, hematogenous metastasis is widely accepted by scholars. Once intestinal metastasis from choriocarcinoma occurs, an experienced gynecological oncologist should be consulted to make a comprehensive judgment based on the patient's vital signs, β-hCG level, and location of the lesion so that an appropriate treatment plan can be developed.

## Data Availability

Data sharing is not applicable to this article as no datasets were generated or analyzed during the current study.

## Abbreviations

hCG: Human chorionic gonadotropin
TVS: Transvaginal color Doppler ultrasound
CT: Computed tomography
Hb: Hemoglobin
CA125: Carbohydrate antigen 125
CEA: Carcinoembryonic antigen
CTA: Computed tomographic angiography
CRP: C-reactive protein
ESMO: European Society for Medical Oncology
GTN: Gestational trophoblastic neoplasia
STRS: Short tandem repeat sequence
HE: Hematoxylin and eosin
